# Resolution of TLR2-induced inflammation through manipulation of metabolic pathways in Rheumatoid Arthritis

**DOI:** 10.1038/srep43165

**Published:** 2017-02-22

**Authors:** Trudy McGarry, Monika Biniecka, Wei Gao, Deborah Cluxton, Mary Canavan, Siobhan Wade, Sarah Wade, Lorna Gallagher, Carl Orr, Douglas J. Veale, Ursula Fearon

**Affiliations:** 1 Molecular Rheumatology, School of Medicine, Trinity Biomedical Sciences Institute, Trinity College Dublin, Dublin 2, Ireland; 2 Centre for Arthritis and Rheumatic Diseases, St. Vincent’s University Hospital, Dublin, Ireland; 3 School of Biochemistry and Immunology, Trinity Biomedical Sciences Institute, Trinity College Dublin, Dublin 2, Ireland

## Abstract

During inflammation, immune cells activated by toll-like receptors (TLRs) have the ability to undergo a bioenergetic switch towards glycolysis in a manner similar to that observed in tumour cells. While TLRs have been implicated in the pathogenesis of rheumatoid arthritis (RA), their role in regulating cellular metabolism in synovial cells, however, is still unknown. In this study, we investigated the effect of TLR2-activation on mitochondrial function and bioenergetics in primary RA-synovial fibroblast cells (RASFC), and further determined the role of glycolytic blockade on TLR2-induced inflammation in RASFC using glycolytic inhibitor 3-(3-pyridinyl)-1-(4-pyridinyl)-2-propen-1-one (3PO). We observed an increase in mitochondrial mutations, ROS and lipid peroxidation, paralleled by a decrease in the mitochondrial membrane potential in TLR2-stimulated RASFC. This was mirrored by differential regulation of key mitochondrial genes, coupled with alteration in mitochondrial morphology. TLR2-activation also regulated changes in the bioenergetic profile of RASFC, inducing PKM2 nuclear translocation, decreased mitochondrial respiration and ATP synthesis and increased glycolysis:respiration ratio, suggesting a metabolic switch. Finally, using 3PO, we demonstrated that glycolytic blockade reversed TLR2-induced pro-inflammatory mechanisms including invasion, migration, cytokine/chemokine secretion and signalling pathways. These findings support the concept of complex interplay between innate immunity, oxidative damage and oxygen metabolism in RA pathogenesis.

The increased proliferation and rapid activation of immune cells during inflammation requires a switch in cell metabolism from a resting regulatory state to a highly metabolically active state, in order to maintain energy homeostasis^1^. This metabolic shift occurs when oxygen levels are low, limiting the metabolism of pyruvate by the tricarboxylic (TCA) cycle in the mitochondria during oxidative phosphorylation. We now know that this metabolic shift occurs in many inflammatory conditions such as colitis, diabetes, psoriasis and obesity^2^,^3^,^4^.

In rheumatoid arthritis (RA) one of the earliest events in synovial inflammation is new vessel formation (angiogenesis), resulting in a self-perpetuating and persistent infiltration of leukocytes, resulting in synovial membrane (SM) hyperplasia^5^,^6^,^7^. The architecture of the microvasculature is highly dysregulated, thus efficiency of oxygen supply to the synovium is poor^6^,^7^. This results in an hypoxic joint microenvironment *in-vivo*, which is associated with increased synovial inflammation, dysfunctional vascularity and activation of key pro-inflammatory signalling mediators such as hypoxia inducible factor 1α (HIF-1α), nuclear factor kappa-light-chain-enhancer of activated B cells (NFĸB), Notch and phosphorylated signal transducer and activator of transcription 3 (STAT3)^7^,^8^,^9^,^10^,^11^,^12^,^13^,^14^. We and others have shown that under hypoxic conditions, cells can switch from mitochondrial respiration to anaerobic glycolysis to satisfy their energy demands in the absence of oxygen, leading to the transcriptional activation of a many glycolytic genes^15^,^16^,^17^. This is consistent with studies showing elevated lactate levels with diminished glucose in RA synovial fluid^18^,^19^,^20^. Studies have also shown increased mitochondrial DNA mutation frequency and mitochondrial dysfunction in the RA joint^21^, effects that are associated with oxidative stress, angiogenesis, pro-inflammatory cytokines and activation of the NLRP3 inflammasome^22^,^23^,^24^,^25^. Furthermore, in RA, studies have shown increased NADPH oxidase/Nox2 in circulating leukocytes and synovium^26^, altered expression of 6-phosphofructo-2-kinase/fructose-2, 6-bisphosphatase 3 (PFKFB3) in naive CD4 T cells^27^, and synovial deficiency of cytochrome C oxidase which is the most commonly recognised respiratory chain defect^24^.

The innate immune system acts as a first line defence against infection, and can initiate downstream signalling pathways, through toll-like receptors (TLRs), resulting in the activation of anti-microbial pro-inflammatory immune responses. Following activation by TLRs, immune cells have the ability to switch to glycolysis in a similar manner to a tumour cell^28^,^29^. Krawczyk *et al*.^29^, described this phenomenon in dendritic cells (DC), with TLR4, TLR2 and TLR9 signalling inducing an increase in glycolytic rate and glucose consumption, promoting expression of glucose transporter Glut1 and lactate production^29^, an effect mediated by phosphoinositide 3-kinase (PI3K) and AMP-activated protein kinase (AMPK) signalling pathways^30^. In macrophages, activation of TLR4, TLR2 and TLR6 promotes an M1 phenotype, resulting in an increase in mitochondrial reactive oxygen species (ROS) and a dependency on glycolysis^31^. Furthermore, certain activated immune cells including dendritic cells, macrophages and T cells, particularly cytotoxic T lymphocytes and T helper 17 (Th17) cells, show Warburg metabolism and HIF-1α induction even under normoxic conditions^17^,^32^. Additionally, HIF-1α has been shown to regulate the murine Th17-Treg axis by promoting Th17 and inhibiting regulatory T cell (Treg) cell differentiation^32^. Furthermore, recent data has shown a link between TLR signaling and HIF-1α activity, with TLR2/4 inducing the activity of HIF-1α under normoxic conditions in human monocyte-derived DC^33^.

While no studies have shown a direct link between TLRs and mitochondrial dysfunction in RA, several studies have demonstrated that TLR signalling is closely associated with the mitochondria in other cell types and diseases. TLR3 signalling induces mitochondrial dysfunction leading to apoptosis in human hepatocytes and the TLR adaptor protein sterile α and heat armadillo-motif-containing protein (SARM) can localise to the mitochondria and trigger intrinsic apoptosis by generation of ROS and depolarisation of the mitochondrial membrane^34^,^35^. Lipopolysaccharide (LPS) stimulation strongly increases the TCA cycle intermediate succinate and reduces the expression of a number of mitochondrial enzymes involved in the TCA cycle^22^,^28^, and citrate released from the mitochondria in response to LPS produces acetyl-CoA^36^. TLR2 and TLR4 signalling regulates mitochondrial biogenesis through the transcription factors NFĸB, cAMP response element-binding protein (CREB) and interferon response factors (IRF-3, IRF-7)^37^. Reciprocally, mitochondrial regulator of fission and morphology protein, MARCH5, is an essential and positive modulator of TLR7 signalling and the mitochondrial protein, ECSIT (evolutionary conserved signalling intermediate in Toll pathways), part of complex I of the electron transport chain (ETC) binds to the TLR signalling protein TNF receptor associated factor protein 6 (TRAF6)^38^.

In this study, we demonstrate alterations in mitochondrial function in the RA joint in response to TLR2 signalling, which is paralleled by dysregulation of glucose metabolism. In addition, we show an increase in glycolytic markers in RA compared to non-inflamed tissue, and a bioenergetics switch in TLR2-treated cells in favour of glycolysis. Furthermore, we show, for the first time, that targeting glycolysis reduces key features of RA pathogenesis *in vitro* and can mediate TLR2-induced inflammation.

## Results

### TLR2 activation induces mitochondrial mutations in RASFC *in vitro*

**Figure 1:**
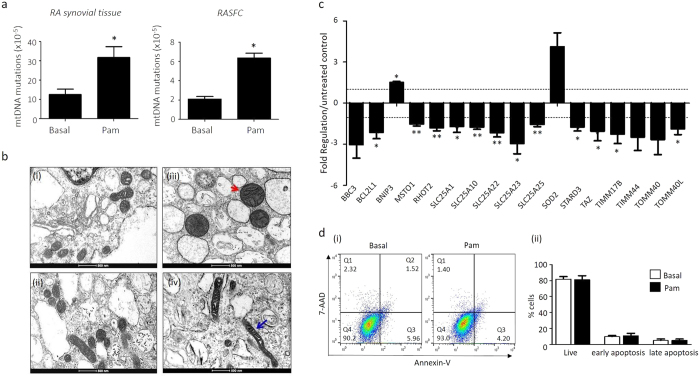
TLR2 activation induces mitochondrial mutations in RASFC *in vitro.* (**a**) Bar graphs representing increased mtDNA mutation frequency in RA *ex vivo* explants (n = 7) and RASFC (n = 6) in response to Pam3CSK4 (1 μg/ml) stimulation for 24 hrs. (**b**) Representative transmission microscopy images of RASFC mitochondria under basal or Pam3CSK4 (1 μg/ml) stimulated conditions. Regular shaped mitochondria are seen in resting cells, in contrast to larger more dense (red arrows) mitochondria and a higher frequency of elongated mitochondria (blue arrow). Scale bar represents 500 nm. (**c)** Bar graph demonstrating 17 genes dysregulated in response to 24 hr incubation with Pam3CSK4 (1 μg/mL) as assessed by RT^2^ PCR Profiler Microarray (n = 3). Dashed line represents no change (1). Data is expressed as up- or down-regulation compared to basal control. Data is normalised to housekeepers: β-Actin, B2M, GAPDH, HPRT1 and RPLP0. (**d**) (i) Representative scatter plots demonstrating Annexin-V/7-AAD double staining in RASFC in response to Pam3CSK4 (1 μg/ml) stimulation for 24 hrs and (ii) representative bar graphs showing percentage of live, early apoptotic and late apoptotic cells under basal control or Pam3CSK4 stimulated conditions (n = 3). Non-parametric data was analysed using Wilcoxin Signed Rank test and parametric data was analysed using paired t-test and is represented as Mean ± SEM, *p < 0.05, **p < *0.01* significantly different to control.

Pam3CSK4-induced mitochondrial damage was assessed using a Random Mutation Capture assay, which screens and detects the presence of random mitochondrial point mutations in the gene encoding the 12 S rRNA subunit. Primary RA synovial fibroblast cells (RASFC) and RA *ex vivo* synovial explants were cultured with Pam3CSK4 for 24 hrs and analysed for the frequency of mitochondrial DNA (mtDNA) mutations and mitochondrial dysfunction. Pam3CSK4 significantly increased mtDNA mutations in both RA synovial tissue and RASFC (Fig. 1a). RA synovial tissue mutations were increased 2.5 fold from a frequency of 1.28 × 10^−5^ to 3.2 × 10^−5^ (p = 0.046) and RASFC mutations were increased 3 fold from a frequency of 2.1 × 10^−5^ to 6.3 × 10^−5^ (p = 0.031).

Using transmission electron microscopy, we next examined the ultrastructure of the RASFC under basal control and Pam3CSK4-stimulated conditions (Fig. 1b). Regularly oval shaped mitochondrial morphology was observed under basal conditions. In contrast, a mixture of irregular and regular shaped mitochondria were observed under TLR2 stimulated conditions. Pam3CSK4 stimulated RASFC tended to have larger (red arrows) and darker mitochondria with a greater frequency of elongated mitochondria (blue arrows), suggesting alteration in mitochondrial function.

RT^2^ Human Mitochondrial Profile PCR Microarrays were used to analyse the expression of 84 human mitochondrial genes in RASFC *in vitro* in response to TLR2 stimulation. Figure 1c demonstrates significant changes in gene expression in basal vs Pam3CSK4-treated RASFC. Seventeen gene targets that are associated with reegulation of mitochondrial function and energy metabolism were identified to be differentially expressed between basal and Pam3CSK4-treated RASFC. Table 1 highlights the 17 dysregulated genes, associated p-values and functions. Of the 17 genes, 15 of these were down-regulated with 2 genes upregulated following Pam3CSK4 stimulation. BCL2/adenovirus E1B 19 kDa-interacting protein (BNIP3) and Superoxide dismutase 2 (SOD2) were increased 1.5 and 4.1 fold, respectively. BCL2 binding component 3 (BBC3), BCL2-like 1 (BCL2L1), Misato homolog 1 (MSTO1), Ras homolog gene family member T2 (RHOT2), Solute carrier family 25 (SLC25) members A1, A10, A22, A23, A25, Star-related lipid transfer domain containing 3 (STARD3), Tafazzin (TAZ), Translocase of inner mitochondrial membrane (TIMM) members 117B and 44 and translocase of outer mitochondrial membrane (TOMM) family members 40 and 40 L were all downregulated in response to Pam3CSK4 (Table 1; Fig. 1c).

To assess if TLR2-inducued mitochondrial dysfunction induces apoptosis in RASFC, an apoptosis assay was performed. Figure 1d (i) demonstrates representative scatterplots of Annexin V-450/7-AAD double staining in RASFC in basal control and TLR2-stimulated cells. In RASFC treated with Pam3CSK4, the population of both early apoptotic cells (Annexin + /7-AAD−) and late apoptotic cells (Annexin + /7-AAD+) are similar to basal control cells (Fig. 1d (ii)), indicating that although TLR2-stimulated RASFC are dysfunctional, they are still resistant to apoptosis.

### TLR2 activation induces mitochondrial dysfunction in RASFC *in vitro*

**Figure 2:**
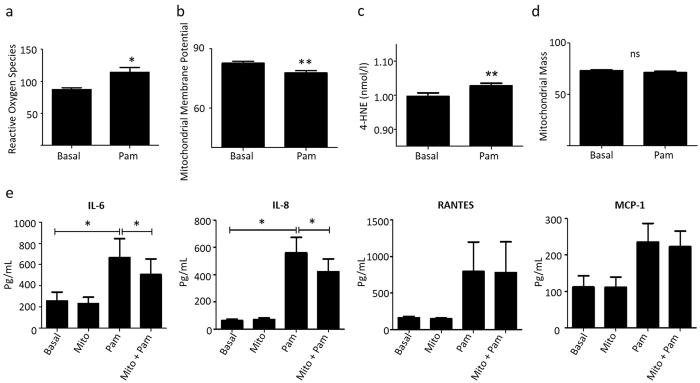
TLR2 activation induces mitochondrial dysfunction in RASFC *in vitro.* (**a**) Representative bar graphs demonstrating the effects of Pam3CSK4 (1 μg/ml) stimulation (24 hrs) on reactive oxygen species (ROS), (**b**) mitochondrial membrane potential (MMP), (**c**) 4-hydroxynonenal (4-HNE) and (**d**) mitochondrial mass (all n = 8). (**e**) Representative bar graphs demonstrating IL-6, IL-8, RANTES and MCP-1 secretion from RASFC following Pam3CSK4 (1 μg/ml) stimulation in the presence or absence of ROS scavenger MitoTEMPO (10 μM) (24 hrs) (n = 5). Data was analysed using Wilcoxin Signed Rank test and is represented as Mean ± SEM, *p < 0.05, **p < *0.01* significantly different to control.

To elucidate if Pam3CSK4-induced mitochondrial mutagenesis and gene alteration in RASFC was accompanied by alterations in mitochondrial function. ROS, mitochondrial membrane potential (MMP) and mitochondrial mass (MM), surrogates of mitochondrial function, were assessed. Pam3CSK4 significantly induced RASFC ROS (p = 0.015) (Fig. 2a) and decreased MMP (p = 0.0019) (Fig. 2b), with no effect observed for MM (p = 0.10) (Fig. 2d). Furthermore, TLR2 significantly induced levels of 4-hydroxynonenal (4-HNE) *in vitro,* a marker of lipid peroxidation, in RASFC (p = 0.002) (Fig. 2c). To further assess the role of ROS on TLR2-induced cytokine and chemokine secretion, ROS scanvenger MitoTEMPO was utilised. TLR2-induced secretion of both IL-6 and IL-8 were significantly reduced in the presence of MitoTEMPO (Fig. 2e). No effect was observed for Rantes or MCP-1.

### The RA synovium is more metabolically active that OA *in vivo*

**Figure 3:**
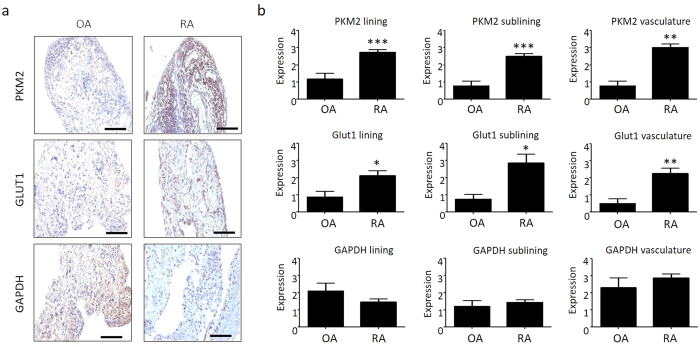
The RA synovium is more metabolically active than OA *in vivo*. (**a**) Representative photomicrographs demonstrating PKM2, Glut1 and GAPDH expression in RA (n = 25) and OA (n = 12) synovium. Mag10X for PKM2, Glut1, GAPDH. Scale bars in the bottom right hang corner represent 100 μm. (**b**) Representative bar graphs demonstrating semi-quantification of immunohistochemical staining of glycolytic markers PKM2, Glut1 and GAPDH in RA (n = 25) and OA (n = 12) lining, sublining and vasculature layers of synovial tissue. Data was analysed using Mann Whitney test and is represented as Mean ± SEM, *p < 0.05, **p < 0.01, ***p < *0.005* significantly different to control.

Synovial expression for markers of glycolysis and oxidative phosphorylation were quantified by immunohistology in both RA and osteoarthritis (OA) patients. Representative images and semi-quantification of glycolysis and oxidative phosphorylation markers are shown in Fig. 3. Pyruvate kinase M2 (PKM2) is more highly expressed in the lining layer (1.16 ± 0.33 OA vs 2.74 ± 0.14 RA), sublining layer (0.78 ± 0.28 OA vs 2.5 ± 0.16 RA) and vasculature (0.78 ± 0.28 OA vs 3.02 ± 0.19 RA) (all p < 0.005) of RA synovial tissue compared to OA. Similarly, Glut1 is more highly expressed in the lining layer (0.87 ± 0.31 OA vs 2.12 ± 0.28 RA), sublining layer (0.37 ± 0.14 OA vs 1.43 ± 0.25 RA) (both p < 0.05) and vasculature (0.5 ± 0.26 OA vs 2.26 ± 0.29 RA) (p < 0.01). No change in synovial expression of glyceraldehyde 3-phosphate dehydrogenase (GAPDH) was observed in RA compared to OA in the lining layer (2.1 ± 0.46 OA vs 1.45 ± 0.18 RA) (p = 0.232), sublining layer (1.20 ± 0.33 OA vs 1.44 ± 0.14 RA) (p = 0.388) and peri-vasculature regions (2.30 ± 0.58 OA vs 2.88 ± 0.23 RA) (p = 0.612).

### TLR2 activation induces bioenergetic changes in RASFC *in vitro*

**Figure 4:**
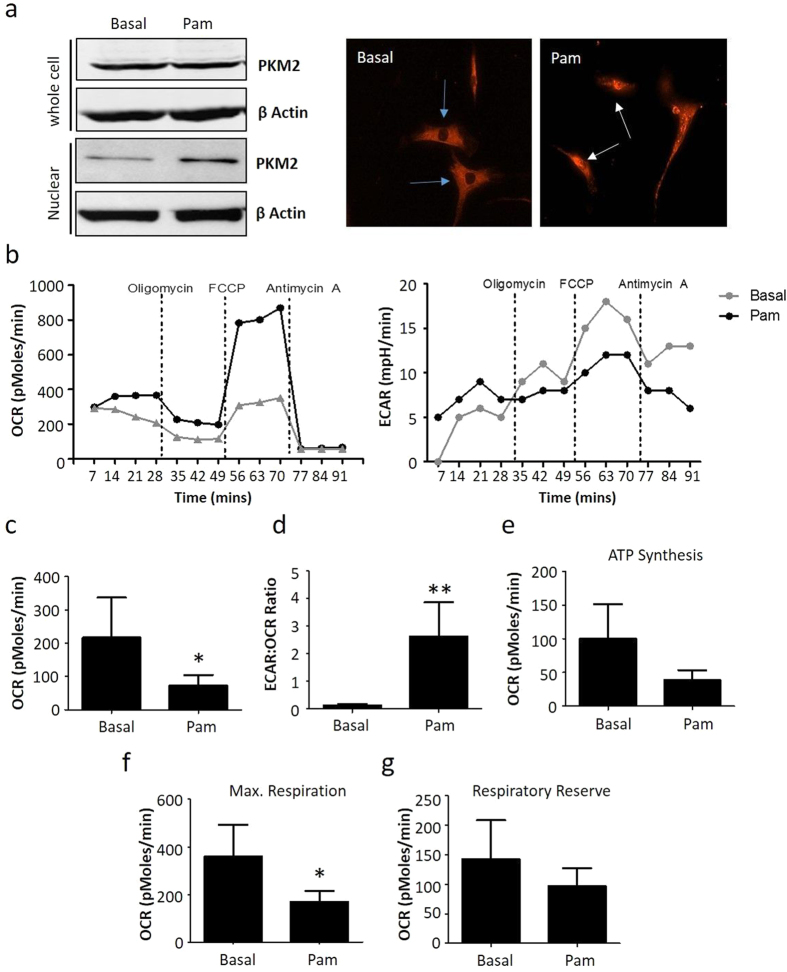
TLR2 activation induces bioenergetics changes in RASFC *in vitro*. (**a**) (i) Representative cropped western blot demonstrating expression of PKM2 in RASFC whole cell lysates and nuclear fragments in response to Pam3CSK4 (1 μg/ml) stimulation (n = 3). (ii) Representative photomicrographs demonstrating Pam3CSK4-induced nuclear localisation of PKM2 as indicated by white arrows. Blue arrows represent lack of staining in nuclei under basal conditions. Mag 20X. (n = 3) (**b**) Representative oxidative phosphorylation (oxygen consumption rate (OCR)) and glycolysis (extracellular acidification rate (ECAR)) Seahorse bioenergetics profiles before and after injections of oligomycin, FCCP and antimycin A for RASFC in the presence and absence of Pam3CSK4 (1 μg/ml). Bar graphs demonstrating (**c**), baseline respiration (OCR), (**d**) the ratio of glycolysis: oxidative phosphorylation (ECAR:OCR), (**e**) ATP synthesis, (**f**) maximal respiration and, (**g**) the respiratory reserve in RASFC in response to Pam3CSK4 (1 μg/ml) stimulation (n = 7). Data was analysed using Wilcoxin Signed Rank test and is represented as Mean ± SEM, *p < 0.05, **p < *0.01* significantly different to control.

As PKM2 is significantly expressed in RA synovial tissue compared to OA, we next assessed the effects of TLR2 signalling on PKM2 levels. Figure 4a demonstrates representative western blots of PKM2, where Pam3CSK4 had no effect on the expression of PKM2 in RASFC whole cell lysates, however, induced the expression of PKM2 in the nuclear fragment of RASFC. Full-length blots are represented in Supplementary Figure 1. Using immunofluorescence, we also demonstrated that Pam3CSK4 induces nuclear localization of PKM2. Figure 4a shows representative photomicrographs of RASFC under basal conditions where PKM2 is localized to the cytoplasm with no nuclear staining observed as indicated by blue arrows. This is in contrast to Pam3CSK4-stimulated RASFC in which nuclear staining of PKM2 is intense, demonstrated by white arrows.

To further investigate if the increased mitochondrial dysfunction and mutations in RASFC were due to alterations in energy metabolism, we measured the two major energy pathways, oxidative phosphorylation and glycolysis using the Seahorse XF-Analyzer. Figure 4b illustrates representative bioenergetics profiles of oxidative phosphorylation (oxygen consumption rate (OCR)) and glycolysis (extracellular acidification rate (ECAR)) before and after injections of oligomycin, carbonyl cyanide-p-trifuoromethoxyphenylhydrazone (FCCP) and antimycin A in control and TLR2-stimulated RASFC. Pam3CSK4 significantly reduced mitochondrial respiration (OCR) (p < 0.05) (Fig. 4c). In contrast, the ECAR:OCR ratio was significantly increased (Fig. 4d). The inhibition of OCR was paralleled by a significant reduction in ATP synthesis (p < 0.05) (Fig. 4e), maximal respiration (p < 0.05) (Fig. 4f) and respiratory reserve (p < 0.05) (Fig. 4g).

### TLR2-induces pro-inflammatory processes are inhibited in the presence of glycolytic inhibitor 3PO *in vitro*

**Figure 5:**
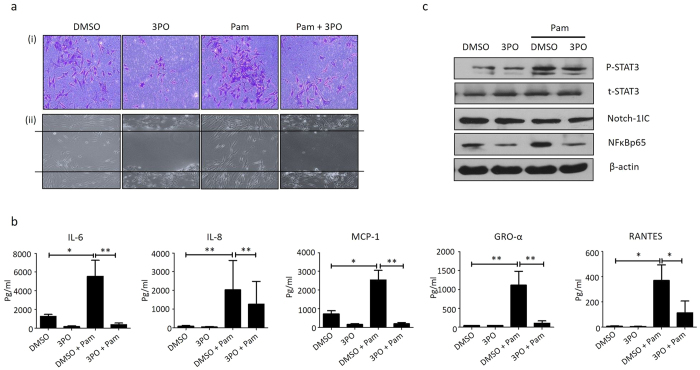
TLR2-induces pro-inflammatory processes are inhibited in the presence of glycolytic inhibitor 3PO *in vitro.* (**a**) Representative images displaying RASFC cell invasion (i) (n = 1) and migration (ii) (n = 3) under Pam3CSK4 (1 μg/ml) stimulation (24 hrs) in the presence of absence of 3PO (20 μM) or DMSO vehicle control. **(b**) Bar graphs demonstrating secretion of IL-6, IL-8, MCP-1, GRO-α and RANTES in RASFC (n = 7) stimulated with Pam3CSK4 (1 μg/ml) for 24 hrs in the presence of absence of 3PO (20 μM) or DMSO vehicle control. (**c**) Representative cropped western blots demonstrating P-STAT3, Notch-1IC and NFĸBp65 expression in K4IM synoviocytes stimulated with Pam3CSK4 (1 μg/ml) in the presence or absence of 3PO (20 μM) or DMSO vehicle control for 24 hrs (n = 1). T-STAT3 was used as a loading control for P-STAT3 and β-actin was used as a loading control for Notch-1IC and NFĸBp65. Data was analysed by Wilcoxin Signed Rank test and is represented as Mean ± SEM, *p < 0.05, **p < *0.01* significantly different to control.

To elucidate if metabolic reprogramming inhibits TLR2-induced pro-inflammatory mechanisms, cells were cultured with glycolytic inhibitor 3PO or DMSO vehicle control in the presence and absence of Pam3CSK4. Figure 5a demonstrates that TLR-2 induced RASFC migration and invasion were inhibited in the presence of 3PO. Furthermore, glycolytic blockade with 3PO inhibited Pam3CSK4-induced IL-6, IL-8, MCP-1, RANTES and GRO-α secretion in both RASFC (Fig. 5b) (all p < 0.05). Finally 3PO alone inhibited NFĸBp65. with no effect observed for Notch-1IC or pSTAT3 (Fig. 5c) and further inhibited Pam3CSK4 induced p-STAT3 and NFĸBp65 (Fig. 5c). Full-length blots are presented in Supplementary Figure 1. This data suggests that 3PO inhibits pro-inflammatory mechanisms in RA and regulates TLR2-induced inflammation and signalling pathways.

## Discussion

In this study, we demonstrate that TLR2 can induce mitochondrial mutations in RA *ex vivo* synovial tissue explants and primary RASFC. We show TLR2 activation significantly induced ROS, 4-HNE and decreased the MMP in RASFC, coupled by changes in ultrastructure of RASFC mitochondria. TLR2-induced mitochondrial dysfunction was further supported by the observed significant changes in expression of key mitochondrial genes involved in apoptosis, transport of proteins into and out of the mitochondria and detoxifying ROS. Mitochondrial dysfunction was paralleled by changes in metabolic activity, with activation of TLR2 significantly reducing mitochondrial respiration, increasing the glycolysis:oxidative phosphorylation ratio and inhibiting ATP synthesis, maximal respiration and respiratory reserve. This was consistent with significant increases *in vivo* in expression of surrogate markers of glycolysis PKM2 and Glut1 in RA synovium compared to OA. TLR2 activation induced translocation of PKM2 into the nucleus in RASFC *in vitro.* We demonstrated that Pam3CSK4-induced chemokine and cytokine secretion, RASFC migration and invasion were inhibited in the presence of glycolytic inhibitor 3PO. Finally, we show that 3PO inhibits TLR2-induced signalling pathways.

In this study, we demonstrate that TLR2 induces random mitochondrial point mutations in RA synovial tissue and primary RASFC when compared to basal control. The mitochondrial genome is highly susceptible to oxidative damage, and has been implicated in disease progression and response to therapy in inflammatory arthritis^39^. This is consistent with previous studies demonstrating increased DNA damage, mtDNA mutation frequency and depolarized mitochondria in RA synovial tissue and peripheral blood mononuclear cells^7^,^21^,^23^,^40^. Furthermore *in vivo* synovial tissue pO2 levels inversely correlated with mitochondrial mutagenesis^24^. The increase in mtDNA mutations both *in vivo* and *in vitro* suggests that accumulation of random mitochondrial mutations in response to TLR2 activation induces a mitochondrial mutator phenotype which may be involved in regulating inflammatory responses.

In the RA joint, ROS levels are a primary source of mitochondrial mutagenesis and dysfunction^41^. In this study, an increase in mtDNA mutation frequency in RASFC in response to TLR2 activation was paralleled by increases in ROS production and 4HNE and decreased MMP. Tumour necrosis factor α (TNFα) can stimulate mtDNA mutations, ROS, MMP and MM in RASFC suggesting direct interplay between mitochondrial function and inflammation^21^. Several TLRs have also been associated with mitochondrial dysfunction and have been shown to induce ROS *in vitro*. LPS induced ROS and hyperpolarization of the mitochondrial membrane potential in human smooth muscle cells (HSMC) and TLR1/2/6 ligands increased ROS in both Raw 264.7 cells and bone-marrow derived macrophages^31^. Studies suggest that this phenomenon is both TLR-specific and cell type specific as while endosomal TLR9 can induce ROS in bone-marrow derived dendritic cells^42^, other studies have shown LPS has no effect^33^.

Lipid peroxidation mediates oxidative stress and is found to be upregulated *in vivo* in several pathological conditions including inflammation, atherosclerosis and chronic degenerative diseases of the nervous system^43^. Previous studies have shown that 4-HNE is highly expressed in the inflamed synovium, is associated with a low *in vivo* oxygen tension and correlates with measures of disease activity including DAS28 and vascularity^23^,^24^. The increase in 4HNE, paralleled by a decrease in MMP is consistent with studies showing that lipid peroxidation can cause the mitochondrial membrane to become more permeable to protons, thus dissipating the MMP^44^, an effect initiated by the reaction of ROS with lipids. In addition, 4-HNE is inhibited in the presence of antioxidants SOD and NAC and *in vivo* levels are decreased in patients following successful TNFα inhibition^24^,^39^. In this study, we observed an increase in 4-HNE in response to TLR2 stimulation. This data taken together suggests that TLR2-induced ROS can induce mitochondrial mutation and lipid peroxidation, increasing 4-HNE and damaging the mitochondrial membrane subsequently decreasing the MMP, further increasing ROS production resulting in a vicious cycle of mitochondrial dysfunction, which can drive inflammation. This is the first study to identify a link between TLR signalling, ROS and mitochondrial dysfunction in RASFC.

In this study we demonstrated altered expression of numerous mitochondrial specific genes in TLR2-activated RASFC *in vitro* including genes associated with apoptosis, redox balance and mitochondrial protein transport. The function of SOD2 in RA is controversial. A recent proteomic study identified that SOD2 was significantly downregulated in 50 primary RASFC lines^45^, implying that inadequate control of ROS is involved in the pathogenesis of RA. Conversely, Chang et al found a 32% increase in SOD2 protein in RA synovial tissue compared to OA and ankylosing spondylitis (AS) tissue^46^. Previous studies in synovial cells have also shown a significant reduction in mitochondrial genome mutagenesis, oxidative stress, altered MMP and transcriptional regulation in the presence of SOD^24^,^47^. In mouse models of arthritis, treatment with SOD reduced pro-inflammatory cytokines^48^, and an increased number of blood vessels has been observed in SOD-1^−/−^ mice^47^. Furthermore, in this study we demonstrated inhibition of TLR2-induced RASFC IL-6 and IL-8 production in the presence of ROS scavenger MitoTEMPO, further supporting a role for an anti-inflammatory effect of anti-oxidants *in vitro*.

The downregulation of apoptotic genes BBC3, BCL2L1 and upregulation of BNIP3 observed in the study are supported by studies showing that RASFC are semi-transformed invasive cells with an aggressive phenotype which is resistant to apoptosis, primarily from inhibition of pro-apoptotic factors including phosphatase and tensin homolog (PTEN), sentrin-specific protease 1 (SEN-P1) and micro RNA-34A and increased expression of pro-survival factors including FLICE-like inhibitory protein (FLIP), small ubiquitin-related modifier 1 (SUMO-1) and overactivity of Ras, Myc and NFĸB pathways^49^,^50^,^51^,^52^. The expression of BNIP3 is strongly activated by hypoxia, is a direct transcriptional target for HIF-1α^53^,^54^, can induce the loss of MMP^55^,^56^. and while upregulated in RA synoviocytes, its cell death activity is dysfunctional^57^. This data, together with the apoptosis assay, suggests that while TLR2 activation can alter mitochondrial function of RASFC, these transformed cells are still resistant to apoptosis, thus maintaining their invasive phenotype. In addition to genes involved in apoptosis, a number of genes coding mitochondrial transporter proteins are dysregulated in response to TLR2 activation. Although there are no studies linking dysregulation of mitochondrial transporters to RA or other auto-immune diseases, a recent study has implicated a family of metal solute carriers (SLCs) in cancer-related transport processes^58^. Solute carriers may have a key role in regulating tumour angiogenesis, cell proliferation and STAT3 signalling in cancer and thus, dysregulation of these genes may also have a role in TLR2-induced pro-inflammatory mechanisms.

Given the observed alterations in synovial mitochondrial function, we next examined the metabolic profile in RA synovial tissue and RASFC. We demonstrated an *in vivo* increase in glycolytic markers PKM2 and Glut1 in RA synovium compared to OA, suggesting that RA synovial tissue is more metabolically active. We also demonstrated a significant change in energy metabolism in response to TLR2 activation, with decreased ATP synthesis, increased nuclear expression of PKM2, decreased mitochondrial respiration and an increased in the ratio of glycolysis: oxidative phosphorylation. This is consistent with studies which have demonstrated an increase in metabolic state towards glycolysis in resident cells in the inflamed joint, such as Th17 cells, macrophages and dendritic cells^38^,^59^. PKM2 is upregulated in tumours and is essential for aerobic glycolysis, it exists as an enzymatically inactive monomer/dimer in the cytosol, which can translocate to the nucleus to activate pro-glycolytic and HIF-1α dependent genes by forming a PKM2-HIF-1α complex. However, PKM2 can also form an enzymatically active tetramer in the cytosol, which limits the nuclear function, destabilizing the PKM2-HIF-1α complex, decreasing glycolytic intermediates and the glycolytic rate^17^,^60^. Studies have also shown increased glycolytic metabolites in RA synovial fluid^18^,^20^,^61^, and RASFC exposed to hypoxic conditions can induce a shift towards glycolysis^15^. Glycolytic metabolites are known to induce many transcriptional factors such as HIF1α and NFĸB, which can induce angiogenic growth factors, inflammatory cytokines and extracellular membrane components, in turn enhancing further glycolytic activity^9^,^22^,^40^,^62^.

To examine if metabolic reprogramming could inhibit pro-inflammatory mechanisms, cells were treated with a glycolytic inhibitor 3PO in the presence or absence of TLR2 activation. Previous studies have shown that 3PO mimics the effects of phosphofructokinase-2/fructose-2,6-bisphosphatase 3 silencing, a key step in the glycolytic pathway^63^. In this study, we demonstrated that 3PO inhibited TLR2-induced RASFC invasion and migration, and several pro-inflammatory cytokines. The effect of 3PO is consistent with previous studies performed in HMVEC which demonstrated its effects on tube formation, vessel sprouting proliferation and migration^63^,^64^,^65^, and can inhibit hypoxia-induced pro-inflammatory pathways in RASFC^15^. Furthermore, 3PO also inhibited TLR2-induced pSTAT3 and NFĸB. Previous studies have shown that TLR2 activation of these pathways plays a crucial role in TLR-induced inflammation^66^,^67^,^68^, with studies showing pSTAT3 inhibition can reduce TLR2-mediated cytokine secretion^67^, and TLR2-induced pro-inflammatory mechanisms are dependent on NFĸB signalling^69^. Therefore depending on the cell type and the inflammatory milieu, 3PO may inhibit STAT3 and NFĸB signalling and subsequent downstream pro-inflammatory mechanisms in RA.

In summary, we have shown that TLR2 activation alters mitochondrial function, increasing ROS which further induces mutations, lipid peroxidation, decreased MMP and a dysregulation of mitochondrial specific genes. This change in mitochondrial function is paralleled by altered cellular bioenergetics, with mitochondrial respiration and ATP synthesis inhibited, increased baseline glycolysis: oxidative phosphorylation ratio and translocation of PKM2 into the nucleus. Metabolic reprogramming with glycolytic inhibitor 3PO may lead to resolution of inflammation, specifically inflammation induced by TLR2. This data further supports the concept of complex interplay between innate immunity, oxidative damage and oxygen metabolism in the pathogenesis of inflammatory arthritis.

## Materials and Methods

### Patient Recruitment, Ethics, Arthroscopy and cell culture

RA patients were recruited from the outpatient clinics at the Department of Rheumatology, St. Vincent’s University Hospital (SVUH). Ethical approval to conduct this study was granted by St. Vincent’s Healthcare Group Medical Research and Ethics Committee and all patients gave fully informed written consent prior to inclusion. All experiments were performed in accordance with these guidelines and regulations. Under local anesthesia, each patient underwent arthroscopy of the inflamed knee, performed using a Wolf 2.7-mm needle arthroscope as previously described^70^. Biopsies were either OCT embedded (TissueTek, Zoeterwoude, The Netherlands) for immunohistochemical analysis or utilized to establish primary RA synovial fibroblasts (RASFC) as previously described^71^. A stable human synoviocyte line (K4IM) from a healthy donor immortalized with SV40 T antigen (TAg) was also utilised^71^,^72^.

### Mitochondrial Random Mutation Capture Assay

To characterize the frequencies of random mutations in RASFC and synovial biopsy samples, we used the mitochondrial Random Mutation Capture assay as described previously^73^. Mitochondrial DNA was extracted using a previously reported protocol^21^. Following extraction, 10 μg of mtDNA was digested with 100 units of *Taq* αI restriction enzyme (New England Biolabs), 1X bovine serum albumin, and a Taq αI–specific digestion buffer (10 m*M* Tris HCl, 10 m*M* MgCl_2,_ 100 m*M* NaCl [pH 8.4]) for 10 hrs, with 100units of *Taq* αI added to the reaction mixture every hour. PCR amplification was performed in 25 μl reaction mixtures containing 12.5 μl 2X SYBR Green Brilliant Mastermix (Stratagene), 0.1 μl uracil DNA glycosylase (New England Biolabs), 0.7 μl forward and reverse primers (10p*M*/μl; IDT), and 6.7 μl H_2_O. The samples were amplified using a Roche LightCycler 480, according to the following protocol; 37 °C for 10 mins, 95 °C for 10 mins, followed by 45 cycles of 95 °C for 15secs and 60 °C for 1 min. Samples were kept at 72 °C for 7 mins and following melting-curve analysis, immediately stored at −80 °C. The primer sequences used were as follows: for mtDNA copy number 5′-ACAGTTTATGTAGCTTACCTCC-3′ (forward) and 5′-TTGCTGCGTGCTTGATGCTTGT-3′ (reverse); for random mutations 5′-CCTCAACAGTTAAATCAACAAAACTGC-3′ (forward) and 5′-GCGCTTACTTTGTAGCCTTCA-3′ (reverse).

### *In vitro* Mitochondrial Dysfunction

Luminescent ATP Detection Assay Kit (Abcam, UK) was used for quantitative detection of total levels of cellular ATP. RASFC were seeded into white 96-wells plate at the density of 1.5 × 10^4^ cells/well and allow attaching overnight. The next day, cells were stimulated with Pam3CSK4 for 24hr. Following cell culture, lysis solution was added into cell suspension to stabilize the ATP. After 5 min, substrate solution was added into each well, shaken for 5 min, and left in the dark for 10 min prior to measurement of luminescence. Cellular ROS Detection Assay Kit (Abcam, Cambridge, UK) was used to determine cellular ROS release and JC-1 Assay Kit (Abcam, UK) and Green-fluuorescent MitoTracker dye (Invitrogen, Ireland) were used to determine MMP and MM of RASFC in the presence of Pam3CSK4, respectively. RASFC were seeded into clear bottom, dark sided 96-wells plate at the density of 2.5 × 10^4^ cells/well and allow attaching overnight. For ROS detection, cells were washed in 1X Buffer and stained with 25 μM DCFDA in 1X Buffer for 45 min at 37 °C and 5% CO_2_. After staining cells were washed, treated with Pam3CSK4 and incubated for 60 min at 37 °C and 5% CO_2._ For MMP, cells were washed in 1X Dilution Buffer and stained with 20 μM JC-1 in 1X Buffer for 10 min at 37 °C and 5% CO_2_. After staining cells were washed, treated with Pam3CSK4 and incubated for 4hr at 37 °C and 5% CO_2._ For the MM, cells were stimulated with Pam3CSK4 for 24hr. Following cell culture, cells were washed with PBS and stained with Green-Fluorescent MitoTracker dye for 45 min at 37 °C and 5% CO_2_. For ROS, MMP and MM, the fluorescence signal was measured using the Spectra Max Gemini.

### Transmission Electron Microscopy

RASFC were grown to confluence in T75 flasks and cultured in the presence of Pam3CSK4 (1 μg/ml) for 24 hrs. RASFC were fixed with gluteraldehyde (3% in 0.05 M Potassium Phosphate buffer, pH 6.8) for 1hr at room temperature. Samples were processed and analyzed using a Jeol JEM2100 LaB6 (operated at 100 Kv). Digital images were obtained using an AMT XR80 capture system and ImageJ software.

### Immunohistochemistry

Immunohistochemistry analysis was performed using 3 μm paraffin synovial tissue sections and the Dako ChemMate Envision Kit (Dako, UK) in RA (n = 25) and OA (n = 12). Sections were baked for 30 mins at 90 °C, deparaffinised in xylene and rehydrated in alcohol and deionised water. Antigen retrieval was performed by heating sections in antigen retrieval solution (15 ml of 1 M sodium citrate and 15 ml of 1 M citric acid in deionised water, pH 6.0) in a pressure cooker. Slides were washed in PBS for 5 mins. Non-specific binding was blocked using 10% casein in PBS for 20 mins. GAPDH (Trevigen, Gaithersburg, MD), PKM2 (Abgent, CA) and GLUT-1 (Abcam, UK) (Santa Cruz Biotechnology, CA) primary antibodies were incubated on sections for 2 hrs at room temperature. An IgG1 control antibody (Dako) was used as a negative control. Slides were incubated for 1hr with horseradish peroxidase–conjugated secondary antibody (Dako). Colour was developed in diaminobenzidine solution (1:50; Dako) and counterstained with hematoxylin. Slides were mounted in Pertex media and analysed using a well-established semi-quantitative scoring method (0–4) and scored separately for perivascular/vascular, lining layer and sub-lining layer regions^74^.

### mRNA extraction and Reverse Transcription to cDNA

RASFC were grown to confluence in T75 flasks and cultured in the presence of Pam3CSK4 (1 μg/ml) for 24 hrs. Total RNA was isolated using RNeasy Mini Kit (Qiagen) according to the manufacturer’s specifications. To ensure sufficient levels of RNA for subsequent PCR arrays, one T75 from two patients were pooled to produce one technical replicate. Three individual arrays were then carried out with a total of 6 donors analysed. The integrity of RNA samples were assessed using a bioanalyzer (Agilent). Samples with a 260:280 nm ratio of 1.8 and above and an RNA integrity number between 7 and 10 were used in subsequent experiments. Isolated RNA was stored at −80 °C. Total RNA (2.5 μg) was reverse transcribed to cDNA using RT^2^ First Strand Kit (Qiagen) as per manufacturer’s instructions. Initially, a genomic elimination step was carried out by adding 2 μL Buffer GE to 2.5 μg RNA and adding RNase-free water to a total volume of 10 μL. This reaction was mixed gently by pipetting up and down, centrifuged briefly, then incubated at 42 °C for 5 min and placed immediately on ice for 1 min to stop the reaction. RNA was added to reverse-transcription mix components (5X Buffer BC3, Control P2, RE3 Reverse Transriptase Mix) and mixed gently by pipetting up and down. This reaction was incubated at 42 °C for 15 min and immediately stopped by incubating at 95 °C for 5 min. 91 μL RNase-free water was added to each reaction and the reaction was either used immediately or stored at −20 °C until the PCR step.

### Mitochondrial Gene Arrays

Human Mitochondria PCR gene microarray was performed using the RT^2^ Profiler™ PCR Array 96 well plates (Qiagen) to simultaneously quantify the expression of 84 mitochondrial genes as per manufacturer’s instruction. Briefly, PCR was performed using the RT^2^ SYBR Green Mastermix (Qiagen). PCR components were mixed in a loading reservoir (1350 μL RT^2^ SYBR Green mastermix, 102 μL cDNA synthesis reaction and 1248 μL RNase-free water) and 25 μL of the reaction was subsequently pipetted into well of the 96 well plate. RT^2^ Profiler™ PCR Arrays included housekeeping genes, negative and internal controls. PCR was performed on a LightCycler 480 System (Roche Diagnostics, Lewes, UK). Thermal cycling conditions were as recommended by the manufacturer (Applied Biosystems). Relative changes in gene expression were determined using the GeneGlobe Data Analysis Centre (Web resource provided by Qiagen) using the 2^−ΔΔCt^ method by normalizing the data to four housekeeping genes: β-Actin, B2M, HPRT1 and RPLP0. Mitochondrial genes included in the array are outlined in Table 2. In order to compare the data between control and treated samples, 2^−ΔΔCt^ values were calculated by normalizing the experimental (Pam3CSK4 treated) data by reference (basal control) data.

### Immunofluorescent Staining for PKM2

RASFC were seeded sparsely onto 8-well chamber slides and serum starved for 24 hrs and subsequently cultured with Pam3CSK4 (1 μg/ml) for a further 24 hrs. Slides were fixed with 1% paraformaldehyde in PBS for 20 min and subsequently incubated with a primary rabbit polyclonal antibody for PKM2 (Cell Signaling Technology) or appropriate IgG control, followed by a 1hr incubation with secondary rabbit antibody-Cy3 (Jackson ImmunoResearch). Stained cells were visualised with a Leitz DM40 microscope (Leica Microsystems, Wetzlar, Germany) and images were captured using the AxioCam system and AxioVision 3.0.6. software (Carl Zeiss Inc., Thornwood, NY, USA).

### Oxygen Consumption Rate and Extracellular Acidification Rate – Seahorse XF24 Technology

Oxygen consumption rate (OCR) and extracellular acidification rate (ECAR), reflecting oxidative phosphorylation and glycolysis, respectively, were measured before and after treatment with oligomycin (2 μg/mL, Seahorse Biosciences, UK), trifluorocarbonylcyanide phenylhydrazone (FCCP) (5 μM, Seahorse Biosciences) and antimycin A (2 μM, Seahorse Biosciences). RASFC were seeded at 30,000 cells per well in 24-well XF-microplates (Seahorse Biosciences) and allowed to adhere. Cells were then subsequently cultured with Pam3CSK4 (1 μg/ml) for 24 hrs. Following this, cells were rinsed in assay medium (unbuffered Dulbecco’s Modified Eagle’s medium (DMEM)) supplemented with 10 mM glucose, pH7.4) before incubation with assay medium for 30 min at 37 °C in a non-CO_2_ incubator. Four baseline OCR and ECAR measurements were obtained over 28 min before injection of specific metabolic inhibitors. Moreover, to challenge the metabolic capacity of the RASFC, three OCR and ECAR measurements were obtained over 15 min following injection with oligomycin, FCCP and antimycin A.

### Apoptosis Assay

RASFC Apoptosis was examined using the Annexin V Apoptosis Deterction Kit eFluor® 450 (eBioscience) according to manufacturer’s instructions. Briefly, RASFC were grown to confluence in 6 well plates and subsequently cultured with Pam3CSK4 (1 μg/ml) for 24 hrs. After this time, cells were scraped, pelleted, washed once with PBS and once with 1X Binding Buffer. Following this, cells were then resuspended in 100 μL 1X Binding Buffer with 5 μL of Annexin V and incubated at RT for 10–15 min. Cells were then washed a final time with 1X Binding Buffer and resuspended in 200 μL of 1X Binding Buffer. Prior to analysis, 5 μL of 7-AAD Viability Staining Solution was added. Cells were analysed using a CyAn ADP Flow Cytometer (Beckman Coulter). Data was analysed using FlowJo software. Cells were electronically gated by their forward scatter, side scatter and doublet exclusion. RASFC were expressed as percentage of live cells (both Annexin V, 7-AAD negative), early apoptotic (Annexin V positive, 7-AAD negative) or late apoptotic cells (both Annexin V, 7-AAD positive).

### Cytokine, Chemokine and 4HNE Measurement

RASFC and HMVEC were seeded in 96-well plates and incubated with Pam3CSK4 (1 μg/ml) in the presence and absence of either ROS scavenger MitoTEMPO (10 μM) or glycolytic inhibitor 3-(3-pyridinyl)-1-(4-pyridinyl)-2-propen-1-one (3PO) (20 μM) or DMSO vehicle control for 24 hr. Supernatants were harvested and IL-6, IL-8, MCP-1, GRO-α,RANTES (R&D systems, UK) and 4HNE (JaICA, Shizuoka, Japan) were measured by specific ELISA according to the manufacturer’s conditions.

### Wound Scratch Assay and Transwell invasion assays

RASFC migration and invasion in response to Pam3CSK4 ± 3PO as previously described^71^,^75^.

### Protein Isolation and Western Blotting

RASFC and K4IM were cultured with Pam3CSK4 (1 μg/ml) a in the presence and absence of 3PO (20 μM) or DMSO vehicle control for 24 hrs prior to lysis in ice-cold RIPA (radio-immunoprecipitation assay) buffer (Sigma-Aldrich). Protein isolation and concentration were performed as previously described^13^. Membranes were incubated with (rabbit polyclonal) anti-PKM2 (1:500:Cell Signalling Technology), rabbit polyclonal anti p-STAT3 (1:500; Cell Signalling Technology), rabbit polyclonal total-STAT3 (1:500; Cell Signalling Technology), rabbit polyclonal anti-Notch-1IC (1:500, Millipore, California, USA), rabbit polyclonal anti-NFĸBp65 (1:1000:Millipore) antibodies (Dilution in PBS containing 0.05% Tween 20 and 2.5% non-fat dried milk at 4 °C overnight with gentle agitation). Anti-mouse monoclonal β-Actin (1:5000; Sigma-Aldrich) was used as a loading control. ECL TM detection reagent (Amersham Biosciences) was placed on the membrane for 5 mins before they were exposed to Hyperfilm ECL.

### Statistical Analysis

SPSS15 system (SPSS Inc, Chicago, Illinois, USA) for windows was used for statistical analysis. Wilcoxon Signed Rank test or Mann Whitney was used for analysis of non-parametric data. Student t-test was used for parametric data. p-values of less than 0.05 (*p < 0.05) were determined as statistically significant.

## Additional Information

**How to cite this article**: McGarry, T. *et al*. Resolution of TLR2-induced inflammation through manipulation of metabolic pathways in Rheumatoid Arthritis. *Sci. Rep.* **7**, 43165; doi: 10.1038/srep43165 (2017).

**Publisher's note:** Springer Nature remains neutral with regard to jurisdictional claims in published maps and institutional affiliations.

## Supplementary Material

Supplementary Information

## Figures and Tables

**Table 1:** TLR2-dysregulated mitochondrial genes in RASFC.

Gene	Up-/Down- Regulation	P Value	*	Function
BBC3	−3.03	0.0544		Belongs to the BH3-only pro-apoptotic proteins. Can induce apoptosis.
BCL2L1	−2.12	0.0217	*	Can act as an anti- or pro-apoptotic protein. Can regulate outer mitochondrial membrane channel opening.
BNIP3	1.51	0.0217	*	Pro-apoptotic protein.
MSTO1	−1.53	0.0030	**	Role in mitochondrial fusion, distribution and morphology.
RHOT2	−1.79	0.0068	**	A RhoGTPase which plays a role in mitochondrial trafficking and fusion-fission dynamics.
SLC25A1	−1.71	0.0232	*	Regulates the movement of citrate across the inner membranes of the mitochondria.
SLC25A10	−1.75	0.0032	**	Provides substrates for metabolic processes including Krebs cycle.
SLC25A22	−2.15	0.0099	**	Mitochondrial glutamate carrier.
SLC25A23	−2.95	0.0341	*	Mitochondrial solute carrier.
SLC25A25	−1.56	0.0038	**	Mitochondrial solute carrier.
SOD2	4.12	0.0907		Destroys superoxide anion radicals.
STARD3	−1.74	0.0109	*	Binds and transports cholesterol.
TAZ	−2.05	0.0494	*	Alters lipids on the inner mitochondrial membrane.
TIMM17B	−2.25	0.0431	*	Facilitates transport of mitochondrial proteins.
TIMM44	−2.47	0.0712		Facilitates ATP-dependant translocation of mitochondrial proteins into the mitochondrial matrix.
TOMM40	−2.66	0.0796		Channel-forming subunit of a complex required for protein import into the mitochondria.
TOMM40L	−1.87	0.0205	*	Role in protein import into the mitochondria.

Table displaying dysregulated genes in response to 24 hrs Pam3CSK4 (1 μg/ml) stimulation. Data is expressed as average up- or down- regulation compared to untreated basal control. ^*^p < 0.05, ^**^p < *0.01* significantly different to control.

**Table 2:** Mitochondrial MicroArrays.

Mitochondrial Genes			
Gene	Gene	Gene	Gene
AIFM2	LRPPRC	SLC25A17	TIMM17A
AIP	MFN1	SLC25A19	TIMM17B
BAK1	MFN2	SLC25A2	TIMM22
BBC3	MIPEP	SLC25A20	TIMM23
BCL2	MPV17	SLC25A21	TIMM44
BCL2L1	MSTO1	SLC25A22	TIMM50
BID	MTX2	SLC25A23	TIMM8A
BNIP3	NEFL	SLC25A24	TIMM8B
CDKN2A	OPA1	SLC25A25	TIMM9
COX10	PMAIP1	SLC25A27	TOOMM20
COX18	RHOT1	SLC25A3	TOMM22
CPT1B	RHOT2	SLC25A30	TOMM34
CPT2	SFN	SLC25A31	TOMM40
DNM1L	SH3GLB1	SLC25A37	TOMM40L
FIS1	SLC25A1	SLC25A4	TOMM70A
TIMM10B	SLC25A10	SLC25A5	TP53
GRPEL1	SLC25A12	SOD1	TSPO
HSP90AA1	SLC25A13	SOD2	UCP1
HSPD1	SLC25A14	STARD3	UCP2
IMMP1L	SLC25A15	TAZ	UCP3
IMMP2L	SLC25A16	TIMM10	UXT

Table displaying list of 84 genes assessed for mitochondrial gene array.

## References

[b1] Chang X. & Wei C. Glycolysis and rheumatoid arthritis. International Journal of Rheumatic Diseases 14, 217–222 (2011). 21816017 10.1111/j.1756-185X.2011.01598.x

[b2] Jiang P., Li H. & Li X. Diabetes mellitus risk factors in rheumatoid arthritis: a systematic review and meta-analysis. Clin. Exp. Rheumatol. 33, 115–21 (2014). 25535750

[b3] Choy E., Ganeshalingam K., Semb A. G., Szekanecz Z. & Nurmohamed M. Cardiovascular risk in rheumatoid arthritis: recent advances in the understanding of the pivotal role of inflammation, risk predictors and the impact of treatment. Rheumatology (Oxford). 53, 2143–54 (2014). 24907149 10.1093/rheumatology/keu224 PMC4241890

[b4] Scrivo R., Vasile M., Müller-Ladner U., Neumann E. & Valesini G. Rheumatic diseases and obesity: adipocytokines as potential comorbidity biomarkers for cardiovascular diseases. Mediators Inflamm. 2013, 808125 (2013). 10.1155/2013/808125 PMC3860141 24376307

[b5] Reece R. J., Canete J. D., Parsons W. J., Emery P. & Veale D. J. Distinct vascular patterns of early synovitis in psoriatic, reactive, and rheumatoid arthritis. Arthritis Rheum. 42, 1481–4 (1999). 10403277 10.1002/1529-0131(199907)42:7<1481::AID-ANR23>3.0.CO;2-E

[b6] Szekanecz Z. & Koch A. E. Endothelial cells in inflammation and angiogenesis. Curr. Drug Targets. Inflamm. Allergy 4, 319–323 (2005). 16101540 10.2174/1568010054022187

[b7] Kennedy A. . Angiogenesis and blood vessel stability in inflammatory arthritis. Arthritis Rheum. 62, 711–721 (2010). 20187131 10.1002/art.27287

[b8] Sivakumar B. . Synovial hypoxia as a cause of tendon rupture in rheumatoid arthritis. J. Hand Surg. Am. 33, 49–58 (2008). 18261665 10.1016/j.jhsa.2007.09.002

[b9] Akhavani M. A. . Hypoxia upregulates angiogenesis and synovial cell migration in rheumatoid arthritis. Arthritis Res. Ther. 11, R64 (2009). 19426483 10.1186/ar2689 PMC2714109

[b10] Ng C. T. . Synovial tissue hypoxia and inflammation *in vivo*. Ann. Rheum. Dis. 69, 1389–1395 (2010). 20439288 10.1136/ard.2009.119776 PMC2946116

[b11] Jeon C. H. . Hypoxia appears at pre-arthritic stage and shows co-localization with early synovial inflammation in collagen induced arthritis. Clin. Exp. Rheumatol. 26, 646–8 (2008). 18799097

[b12] Oliver K. M., Taylor C. T. & Cummins E. P. Hypoxia. Regulation of NFkappaB signalling during inflammation: the role of hydroxylases. Arthritis Res. Ther. 11, 215 (2009). 19291263 10.1186/ar2575 PMC2688226

[b13] Gao W. . Notch signalling pathways mediate synovial angiogenesis in response to vascular endothelial growth factor and angiopoietin 2. Ann. Rheum. Dis. 72, 1080–8 (2013). 23161900 10.1136/annrheumdis-2012-201978 PMC3664379

[b14] Gao W. . Hypoxia and STAT3 signalling interactions regulate pro-inflammatory pathways in rheumatoid arthritis. Ann. Rheum. Dis. 74, 1275–83 (2015). 24525913 10.1136/annrheumdis-2013-204105

[b15] Biniecka M. . Dysregulated bioenergetics: a key regulator of joint inflammation. Ann. Rheum. Dis. annrheumdis –2015–208476, doi: 10.1136/annrheumdis-2015-208476 (2016). PMC5136702 27013493

[b16] Shi L. Z. . HIF1alpha-dependent glycolytic pathway orchestrates a metabolic checkpoint for the differentiation of TH17 and Treg cells. J. Exp. Med. 208, 1367–76 (2011). 21708926 10.1084/jem.20110278 PMC3135370

[b17] Palsson-McDermott E. M. & O’Neill L. A. J. The Warburg effect then and now: from cancer to inflammatory diseases. Bioessays 35, 965–73 (2013). 24115022 10.1002/bies.201300084

[b18] Naughton D. P. . A comparative evaluation of the metabolic profiles of normal and inflammatory knee-joint synovial fluids by high resolution proton NMR spectroscopy. FEBS Lett. 332, 221–5 (1993). 7691662 10.1016/0014-5793(93)80636-9

[b19] Ciurtin C. . Correlation between different components of synovial fluid and pathogenesis of rheumatic diseases. Rom. J. Intern. Med. 44, 171–181 (2006). 17236298

[b20] Hitchon C. A., El-Gabalawy H. S. & Bezabeh T. Characterization of synovial tissue from arthritis patients: A proton magnetic resonance spectroscopic investigation. Rheumatol. Int. 29, 1205–1211 (2009). 19184029 10.1007/s00296-009-0865-z

[b21] Harty L. C. . Mitochondrial mutagenesis correlates with the local inflammatory environment in arthritis. Annals of the Rheumatic Diseases 71, 582–588 (2012). 22121133 10.1136/annrheumdis-2011-200245

[b22] Tannahill G. M. . Succinate is an inflammatory signal that induces IL-1β through HIF-1α. Nature 496, 238–42 (2013). 23535595 10.1038/nature11986 PMC4031686

[b23] Biniecka M. . Oxidative damage in synovial tissue is associated with *in vivo* hypoxic status in the arthritic joint. Ann. Rheum. Dis. 69, 1172–1178 (2010). 19706618 10.1136/ard.2009.111211

[b24] Biniecka M. . Hypoxia induces mitochondrial mutagenesis and dysfunction in inflammatory arthritis. Arthritis Rheum. 63, 2172–2182 (2011). 21484771 10.1002/art.30395

[b25] Zhou R., Yazdi A. S., Menu P. & Tschopp J. A role for mitochondria in NLRP3 inflammasome activation. Nature 469, 221–5 (2011). 21124315 10.1038/nature09663

[b26] Biniecka M. . Redox-mediated angiogenesis in the hypoxic joint of inflammatory arthritis. Arthritis Rheumatol. (Hoboken, N.J.) 66, 3300–10 (2014). 10.1002/art.38822 25155522

[b27] Yang Z., Fujii H., Mohan S. V., Goronzy J. J. & Weyand C. M. Phosphofructokinase deficiency impairs ATP generation, autophagy, and redox balance in rheumatoid arthritis T cells. J. Exp. Med. 210, 2119–34 (2013). 24043759 10.1084/jem.20130252 PMC3782046

[b28] Garedew A., Henderson S. O. & Moncada S. Activated macrophages utilize glycolytic ATP to maintain mitochondrial membrane potential and prevent apoptotic cell death. Cell Death Differ. 17, 1540–1550 (2010). 20339378 10.1038/cdd.2010.27

[b29] Krawczyk C. M. . Toll-like receptor-induced changes in glycolytic metabolism regulate dendritic cell activation. Blood 115, 4742–4749 (2010). 20351312 10.1182/blood-2009-10-249540 PMC2890190

[b30] Dong H. & Bullock T. N. J. Metabolic influences that regulate dendritic cell function in tumors. Front. Immunol. 5, 24 (2014). 24523723 10.3389/fimmu.2014.00024 PMC3906600

[b31] West A. P. . TLR signalling augments macrophage bactericidal activity through mitochondrial ROS. Nature 472, 476–80 (2011). 21525932 10.1038/nature09973 PMC3460538

[b32] Dang E. V. . Control of T(H)17/T(reg) balance by hypoxia-inducible factor 1. Cell 146, 772–84 (2011). 21871655 10.1016/j.cell.2011.07.033 PMC3387678

[b33] Spirig R. . Effects of TLR agonists on the hypoxia-regulated transcription factor HIF-1alpha and dendritic cell maturation under normoxic conditions. PLoS One 5, e0010983 (2010). 20539755 10.1371/journal.pone.0010983 PMC2881864

[b34] Panneerselvam P., Singh L. P., Ho B., Chen J. & Ding J. L. Targeting of pro-apoptotic TLR adaptor SARM to mitochondria: definition of the critical region and residues in the signal sequence. Biochem. J. 442, 263–71 (2012). 22145856 10.1042/BJ20111653

[b35] Djafarzadeh S., Vuda M., Takala J., Ochs M. & Jakob S. M. Toll-like receptor-3-induced mitochondrial dysfunction in cultured human hepatocytes. Mitochondrion 11, 83–8 (2011). 20691286 10.1016/j.mito.2010.07.010

[b36] Infantino V. . The mitochondrial citrate carrier: a new player in inflammation. Biochem. J. 438, 433–6 (2011). 21787310 10.1042/BJ20111275

[b37] Piantadosi C. A. & Suliman H. B. Redox regulation of mitochondrial biogenesis. Free Radic. Biol. Med. 53, 2043–53 (2012). 23000245 10.1016/j.freeradbiomed.2012.09.014 PMC3604744

[b38] Shi H.-X. . Mitochondrial ubiquitin ligase MARCH5 promotes TLR7 signaling by attenuating TANK action. PLoS Pathog. 7, e1002057 (2011). 21625535 10.1371/journal.ppat.1002057 PMC3098239

[b39] Biniecka M. . Successful tumour necrosis factor (TNF) blocking therapy suppresses oxidative stress and hypoxia-induced mitochondrial mutagenesis in inflammatory arthritis. Arthritis Res. Ther. 13, R121 (2011). 21787418 10.1186/ar3424 PMC3239359

[b40] Moodley D., Mody G., Patel N. & Chuturgoon A. A. Mitochondrial depolarisation and oxidative stress in rheumatoid arthritis patients. Clin. Biochem. 41, 1396–401 (2008). 18789914 10.1016/j.clinbiochem.2008.08.072

[b41] Bulua A. C. . Mitochondrial reactive oxygen species promote production of proinflammatory cytokines and are elevated in TNFR1-associated periodic syndrome (TRAPS). J. Exp. Med. 208, 519–33 (2011). 21282379 10.1084/jem.20102049 PMC3058571

[b42] Lahiri A. . TLR 9 activation in dendritic cells enhances salmonella killing and antigen presentation via involvement of the reactive oxygen species. PLoS One 5, e13772 (2010). 21048937 10.1371/journal.pone.0013772 PMC2966436

[b43] Ayala A., Muñoz M. F. & Argüelles S. Lipid peroxidation: production, metabolism, and signaling mechanisms of malondialdehyde and 4-hydroxy-2-nonenal. Oxid. Med. Cell. Longev. 2014, 360438 (2014). 24999379 10.1155/2014/360438 PMC4066722

[b44] Gottlieb R. A. Mitochondria: execution central. FEBS Lett. 482, 6–12 (2000). 11018514 10.1016/s0014-5793(00)02010-x

[b45] Wang J.-G. . Disorders in angiogenesis and redox pathways are main factors contributing to the progression of rheumatoid arthritis: a comparative proteomics study. Arthritis Rheum. 64, 993–1004 (2012). 22006448 10.1002/art.33425

[b46] Chang X. . Identification of proteins with increased expression in rheumatoid arthritis synovial tissues. J. Rheumatol. 36, 872–80 (2009). 19369474 10.3899/jrheum.080939

[b47] Kubota M. . Hydrogen and N-acetyl-L-cysteine rescue oxidative stress-induced angiogenesis in a mouse corneal alkali-burn model. Invest. Ophthalmol. Vis. Sci. 52, 427–33 (2011). 20847117 10.1167/iovs.10-6167

[b48] Iyama S. . Treatment of murine collagen-induced arthritis by *ex vivo* extracellular superoxide dismutase gene transfer. Arthritis Rheum. 44, 2160–7 (2001). 11592381 10.1002/1529-0131(200109)44:9<2160::aid-art369>3.0.co;2-0

[b49] Muller-Ladner U. . Synovial fibroblasts of patients with rheumatoid arthritis attach to and invade normal human cartilage when engrafted into SCID mice. Am J Pathol 149, 1607–15 (1996). 8909250 PMC1865262

[b50] Firestein G. S. & Zvaifler N. J. How important are T cells in chronic rheumatoid synovitis? Arthritis Rheum. 33, 768–73 (1990). 2194461 10.1002/art.1780330602

[b51] Ospelt C., Neidhart M., Gay R. E. & Gay S. Synovial activation in rheumatoid arthritis. Front. Biosci. 9, 2323–34 (2004). 15353290 10.2741/1399

[b52] Korb A., Pavenstädt H. & Pap T. Cell death in rheumatoid arthritis. Apoptosis 14, 447–454 (2009). 19199037 10.1007/s10495-009-0317-y

[b53] Bruick R. K. Expression of the gene encoding the proapoptotic Nip3 protein is induced by hypoxia. Proc. Natl. Acad. Sci. USA 97, 9082–7 (2000). 10922063 10.1073/pnas.97.16.9082 PMC16825

[b54] Kothari S. . BNIP3 plays a role in hypoxic cell death in human epithelial cells that is inhibited by growth factors EGF and IGF. Oncogene 22, 4734–44 (2003). 12879018 10.1038/sj.onc.1206666

[b55] Regula K. M., Ens K. & Kirshenbaum L. A. Inducible expression of BNIP3 provokes mitochondrial defects and hypoxia-mediated cell death of ventricular myocytes. Circ. Res. 91, 226–31 (2002). 12169648 10.1161/01.res.0000029232.42227.16

[b56] Vande Velde C. . BNIP3 and genetic control of necrosis-like cell death through the mitochondrial permeability transition pore. Mol. Cell. Biol. 20, 5454–68 (2000). 10891486 10.1128/mcb.20.15.5454-5468.2000 PMC85997

[b57] Kammouni W. . Regulation of apoptosis in fibroblast-like synoviocytes by the hypoxia-induced Bcl-2 family member Bcl-2/adenovirus E1B 19-kd protein-interacting protein 3. Arthritis Rheum. 56, 2854–63 (2007). 17763440 10.1002/art.22853

[b58] Jong N. N. & McKeage M. J. Emerging roles of metal solute carriers in cancer mechanisms and treatment. Biopharm. Drug Dispos. 35, 450–62 (2014). 24889085 10.1002/bdd.1903

[b59] Végran F., Boidot R., Michiels C., Sonveaux P. & Feron O. Lactate Influx through the Endothelial Cell Monocarboxylate Transporter MCT1 Supports an NF-κB/IL-8 Pathway that Drives Tumor Angiogenesis. Cancer Res. 71 (2011). 10.1158/0008-5472.CAN-10-2828 21300765

[b60] Palsson-McDermott E. M. . Pyruvate Kinase M2 Regulates Hif-1α Activity and IL-1β Induction and Is a Critical Determinant of the Warburg Effect in LPS-Activated Macrophages. Cell Metab. 21, 65–80 (2015). 25565206 10.1016/j.cmet.2014.12.005 PMC5198835

[b61] Ciurtin C. . Correlation between different components of synovial fluid and pathogenesis of rheumatic diseases. Rom. J. Intern. Med. 44, 171–81 (2006). 17236298

[b62] Fearon U., Canavan M., Biniecka M. & Veale D. J. Hypoxia, mitochondrial dysfunction and synovial invasiveness in rheumatoid arthritis. - PubMed - NCBI. Nat. Rev. Rheumatol. 7, 385–97 (2016). 10.1038/nrrheum.2016.69 27225300

[b63] Clem B. . Small-molecule inhibition of 6-phosphofructo-2-kinase activity suppresses glycolytic flux and tumor growth. Mol. Cancer Ther. 7, 110–20 (2008). 18202014 10.1158/1535-7163.MCT-07-0482

[b64] De Bock K. . Role of PFKFB3-driven glycolysis in vessel sprouting. Cell 154, 651–63 (2013). 23911327 10.1016/j.cell.2013.06.037

[b65] Schoors S. . Partial and transient reduction of glycolysis by PFKFB3 blockade reduces pathological angiogenesis. Cell Metab. 19, 37–48 (2014). 24332967 10.1016/j.cmet.2013.11.008

[b66] Luu K. . STAT1 plays a role in TLR signal transduction and inflammatory responses. Immunol. Cell Biol. 92, 761–9 (2014). 25027037 10.1038/icb.2014.51

[b67] Zgheib A., Pelletier-Bonnier É., Levros L.-C. & Annabi B. Selective JAK/STAT3 signalling regulates transcription of colony stimulating factor-2 and -3 in Concanavalin-A-activated mesenchymal stromal cells. Cytokine 63, 187–93 (2013). 23688618 10.1016/j.cyto.2013.04.027

[b68] Toubi E. & Shoenfeld Y. Toll-like receptors and their role in the development of autoimmune diseases. Autoimmunity 37, 183–8 (2004). 15497450 10.1080/08916930410001704944

[b69] Jin J. . Coactivation of TLR4 and TLR2/6 coordinates an additive augmentation on IL-6 gene transcription via p38MAPK pathway in U937 mononuclear cells. Mol. Immunol. 49, 423–32 (2011). 22030478 10.1016/j.molimm.2011.08.026 PMC3224151

[b70] Fearon U. . Oncostatin M induces angiogenesis and cartilage degradation in rheumatoid arthritis synovial tissue and human cartilage cocultures. Arthritis Rheum. 54, 3152–3162 (2006). 17009243 10.1002/art.22161

[b71] Connolly M. . A-SAA regulates TNFalpha and matrix turnover and predicts disease progression in patients pre/post biologic therapy. Arthritis Rheum (2011). 10.1002/art.33455 22076945

[b72] Haas C. S. . Inhibition of angiogenesis by interleukin-4 gene therapy in rat adjuvant-induced arthritis. Arthritis Rheum. 54, 2402–14 (2006). 16869003 10.1002/art.22034

[b73] Vermulst M., Bielas J. H. & Loeb L. A. Quantification of random mutations in the mitochondrial genome. Methods 46, 263–8 (2008). 18948200 10.1016/j.ymeth.2008.10.008 PMC2615251

[b74] Youssef P. P. . Quantitative microscopic analysis of inflammation in rheumatoid arthritis synovial membrane samples selected at arthroscopy compared with samples obtained blindly by needle biopsy. Arthritis Rheum. 41, 663–669 (1998). 9550475 10.1002/1529-0131(199804)41:4<663::AID-ART13>3.0.CO;2-L

[b75] McGarry T. . Toll-like receptor 2 (TLR2) induces migration and invasive mechanisms in rheumatoid arthritis. Arthritis Res. Ther. 17, 153 (2015). 26055925 10.1186/s13075-015-0664-8 PMC4495696

