# Supplementary material for: Resolution of TLR2-induced inflammation through manipulation of metabolic pathways in Rheumatoid Arthritis
Source: Sci Rep. 2017 Feb 22;7:43165. doi: 10.1038/srep43165 (PMC5320554; doi:10.1038/srep43165)
Supplement: Supplementary Information [file srep43165-s1.pdf]

Resolution of TLR2-induced inflammation through manipulation of metabolic pathways in Rheumatoid Arthritis

Trudy McGarry[1,2], Monika Biniecka[2], Wei Gao[2], Deborah Cluxton[3], Mary Canavan[1,2], Siobhan Wade[1], Sarah Wade[1,2], Lorna Gallagher[2], Carl Orr[2], Douglas J. Veale[2], Ursula Fearon[1]*

[1] Molecular Rheumatology, School of Medicine, Trinity Biomedical Sciences Institute, Trinity College Dublin, Dublin 2, Ireland.
[2] Centre for Arthritis and Rheumatic Diseases, St. Vincent's University Hospital, Dublin, Ireland.
[3] School of Biochemistry and Immunology, Trinity Biomedical Sciences Institute, Trinity College Dublin, Dublin 2, Ireland.

Corresponding author:
*Ursula Fearon
Prof. of Molecular Rheumatology
Trinity Biomedical Sciences Centre
Trinity College Dublin
Phone: (01) 896 2659
Email: fearonu@tcd.ie
prtli

Supplementary Figure 1

Full-Length Western Blots. **a** demonstrates full-length western blots of PKM2 and β Actin loading control protein in rheumatoid arthritis (RA) synovial fibroblast cells from 3 RA patients (RA1 – 3) in whole cell lysates under basal control or Pam3CSK4-treated (1 µg/ml) conditions. **b** demonstrates full-length western blots of PKM2 and β Actin loading control protein in basal and Pam3CSK4-stimulated RASFC nuclear and cytoplasmic fragments. **c** demonstrates full-length western blots of Notch1-IC, p-STAT3 and NFκB and loading control proteins total STAT3 and β Actin in whole cell lysates from K4IM synoviocytes stimulated with Pam3CSK4 in the presence and absence of glycolytic inhibitor 3PO (20 µM) or DMSO vehicle control. Far right lane in blots **a – b** demonstrates molecular size marker. Membranes in western blots in **c** were cut prior to incubation with primary antibody.

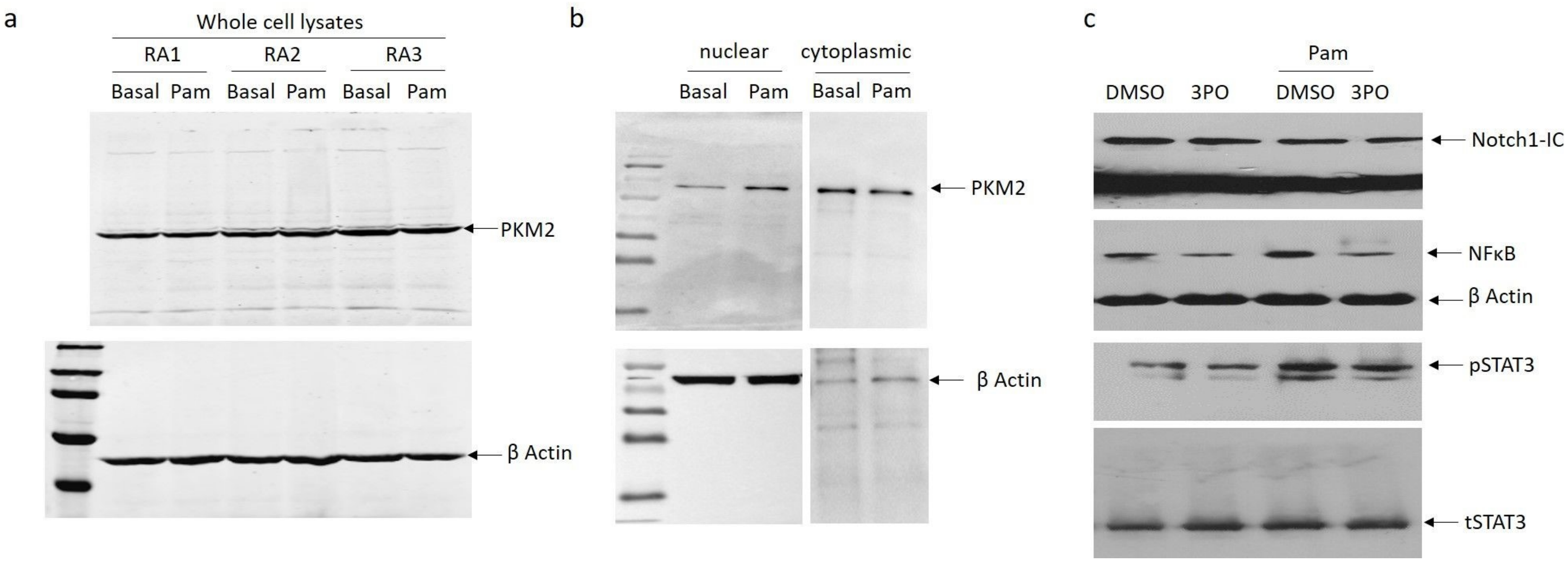
a
Whole cell lysates
RA1
RA2
RA3
Basal
Pam
Basal
Pam
Basal
Pam
PKM2
β Actin
b
nuclear
cytoplasmic
Basal
Pam
Basal
Pam
PKM2
β Actin
c
Pam
DMSO
3PO
DMSO
3PO
Notch1-IC
NFκB
β Actin
pSTAT3
tSTAT3
